# Efficacy of a commercial bacteriophage cocktail against planktonic cells and both thin and thick biofilms of skin pathogens, measured using isothermal microcalorimetry

**DOI:** 10.3389/fmicb.2025.1608243

**Published:** 2025-07-30

**Authors:** Tecla Lafranca, Gernot Bonkat, Malte Rieken, Olivier Braissant

**Affiliations:** ^1^Department of Biomedical Engineering, University of Basel, Basel, Switzerland; ^2^alta uro AG, Basel, Switzerland

**Keywords:** phage (bacteriophage), isothermal calorimetry, biofilms, *Staphylococcus*, phage therapies

## Abstract

**Introduction:**

Skin and soft tissue infections are frequent and often require antibiotic treatment. However, for mild and self-limiting lesions, bacteriophage therapy could be an interesting treatment option that limits the use of antimicrobials and helps avoid the development of resistance. Still, very little is known about the efficacy of commercial phage cocktails against the biofilms encountered in these lesions. In this study, we investigated the use of a commercial phage cocktail against Staphylococci and Streptococci grown planktonically in thin and thick biofilms.

**Methods:**

Isothermal microcalorimetry was used to monitor the metabolic activity of planktonic cells, as well as cells grown in thin or thick biofilms of common skin pathogens (*Staphylococcus aureus*, *Staphylococcus epidermidis*, and *Streptococcus agalactiae*), when exposed to the commercial phage cocktail.

**Results:**

The use of phages against sensitive strains showed a rapid decrease in metabolic activity in planktonic cells. However, when applied to a thin biofilm, the effect was already less, although it was still important. Finally, no effect was visible on thick and mature biofilms.

**Conclusion:**

The efficacy of bacteriophage cocktails is limited by the thickness and maturation of biofilms. In the case of skin and soft tissue infections, especially for chronic wounds, it might be necessary to mechanically remove and disrupt the biofilm through mechanical debridement to enable the phage product to be effective.

## Introduction

Skin and soft tissue infections (SSTIs) present a broad clinical spectrum, ranging from mild, self-limiting lesions, such as cellulitis, to serious, life-threatening conditions such as necrotizing fasciitis (Hatlen and Miller, 2021). The foot and lower leg are the most frequently affected body parts, particularly in diabetic patients (Shittu and Lin, 2006). A large proportion of patients require medical treatment and hospitalization, and in some cases, the outcome is fatal (Moffarah et al., 2016). Current treatment options involve antibiotics and, if necessary, surgery (Hatlen and Miller, 2021). The bacterium responsible for most SSTIs is *Staphylococcus aureus* (*S. aureus*), which is a ubiquitous gram-positive bacterium (Hatlen and Miller, 2021). It is generally part of the natural flora but might become an opportunistic pathogen (Paharik and Horswill, 2016). *Staphylococcus aureus* is also the leading cause of various other serious infections, including bacteremia, meningitis, endocarditis, osteomyelitis, and pneumonia (Tong et al., 2015), and a leading cause of both hospital- and community-acquired infections worldwide (Lowy, 1998; Whittard et al., 2021).

Increasing evidence has confirmed the importance of biofilms in various skin conditions, such as diabetic and venous stasis ulcers, necrotizing fasciitis, pressure ulcers, cellulitis, atopic dermatitis, erythema nodosum, and erysipelas (Severn and Horswill, 2023). Indeed, biofilm formation in chronic wounds inhibits healing by delaying re-epithelialization (Schierle et al., 2009). Biofilms are communities of micro-organisms embedded in a matrix of polymeric substances characterized by three major growth phases: (1) attachment to the surface [in our field, e.g., on the soft tissues or the epidermal layer of the skin (Kumar et al., 2019)], (2) maturation, and (3) dispersion. Biofilms act as a protective layer against hostile physical and chemical hostile conditions, including antibacterial drugs. The induction and spread of resistant strains, such as methicillin- or vancomycin-resistant *S. aureus* (MRSA or VRSA) (Whittard et al., 2021), are of increasing concern. The continuous emergence of new resistance not only against penicillin but also against new agents such as linezolid (Tsiodras et al., 2001) highlights an urgent need to find new effective therapies. This situation necessitates increasing the antimicrobial dosage in response to rising minimal inhibitory concentration (MIC) or combining multiple antibiotics (Presterl et al., 2009).

Bacteriophage therapy is an option already used in some countries and is gaining popularity in Europe. Bacteriophages, or phages, are viruses that use bacterial hosts for replication, ultimately leading to the destruction of these bacteria (Kiani et al., 2021; Kim et al., 2021; Gündoǧdu et al., 2016). Their discovery dates back to 1915, but after the discovery of penicillin, interest in phages waned in Western Europe and is only now resurfacing due to the emergence of antibiotic resistance (Clokie et al., 2011). However, in Russia and Georgia, research has been ongoing, and phage products are used in clinical settings and readily found in online pharmacies (Clokie et al., 2011). Western European agencies, on the other hand, require further research into these products; thus, phage therapy has not yet been accepted (Gordillo Altamirano and Barr, 2019).

Products found online are often a mix of different phage strains (or phage types) and are known as a cocktail. Indeed, phages exhibit high host specificity; therefore, using several phages in the same product broadens the spectrum of activity and increases the chance of therapy being efficient. It is also hypothesized to reduce the emergence of resistant variants (Chan and Abedon, 2012). For example, the cocktail used in this study (see later), called “Fersisi,” can be found in an online pharmacy. The indications for such a product are broad, ranging from skin infections to otolaryngological diseases, surgical diseases, inflammations of the oral cavity, eye diseases, secondary infections of thermal burns, urogenital and gynecological infections, or enteric infections.^1^ Although Russian literature on the use of phages cannot be assessed in detail, the results obtained to date are promising (Morozova et al., 2018). Phages appear to be a valid alternative to antibiotics (Jiang et al., 2021), as they are effective against several bacteria, including *S. aureus* in skin diseases (Kiani et al., 2021), and prevent or reduce the formation of their biofilm (Tan et al., 2020; Kelly et al., 2012; Alves et al., 2014). It has also been shown that the bacteria’s virulence is decreased as sensitivity to certain antibiotics increases through the use of phages (León and Bastías, 2015). Similarly, combining phages with anitibiotics has been shown to be even more effective (Jiang et al., 2021; Łusiak-Szelachowska et al., 2020). However, the lack of validated clinical trials poses a challenge that needs to be addressed in the coming years (Parracho et al., 2012).

Analysis and testing of commercial phage products remain a challenge as culture-based conventional methods require a significant amount of time, resources, and laboratory staff. Similarly, optical density-based methods are also limited because some processes cannot be distinguished from others. Moreover, such methods perform poorly on solid and/or opaque substrates or media, making assessment of anti-biofilm activity an even bigger challenge. Therefore, this study utilizes an isothermal microcalorimeter (IMC) to investigate the use of these cocktails against *S. aureus* and assess the metabolic activity of planktonic cells, as well as thin and thick biofilms. IMC records the metabolic heat produced during bacterial growth in real-time, resulting in a heat flow curve that is directly comparable to the metabolic activity (see details in Bonkat et al., 2012; Nykyri et al., 2019; Braissant et al., 2020). IMC has been previously used to investigate the growth of Staphylococci and the effect of phages. For example, Molendijk et al. (2023) used different methods, including IMC, to assess the susceptibility of *S. aureus* to phage mixes and single phages. The antimicrobial efficacy and antibiofilm activity of phages against *Staphylococcus epidermidis* (*S. epidermidis*) were also demonstrated using IMC (Fanaei Pirlar et al., 2022). Similarly, the effect of antibiotics or phages on *S. aureus* biofilms was also investigated using a microcalorimeter (Butini et al., 2019; Sultan et al., 2022). This study aimed to analyze the effect of bacteriophages on planktonic cells in liquid cultures, on thin biofilms previously grown in a well plate calorimeter, and on thick biofilms using semi-permeable filters that can be easily transferred to fresh medium to allow further growth (Solokhina et al., 2018; Merritt et al., 2011; Solokhina et al., 2019).

## Materials and methods

### Microorganisms and phage products used

The bacteriophage used was the Fersisi (Eliava Biopreparations, Tbilisi, 0160, Georgia). The product was obtained from an online pharmacy as a box of five ampoules of phages with titers against Staphylococci (*S. aureus*, *S. epidermidis*) of no <10^5^ mL^−1^, and titers against Streptococci (*S. pyogenes*, *S. sanguis*, *S. salivarius*, *S. agalactiae*) of no <10^4^ mL^−1^ (as indicated by the manufacturer). The Fersisi bacteriophage cocktail was described in detail through previous metagenomic analysis (McCallin et al., 2018). *S. aureus* (ATCC 29213, ATCC 43300, and ATCC 25923), *S. epidermidis* (ATCC 49461), and *S. agalactiae* (DSM 6784) were obtained from the ATCC culture collection.

### Calorimetry of liquid culture

The bacterial strains used were stored at −80°C. Before each experiment, purity was visually checked after overnight culture on agarized brain-heart infusion (BHI composition: calf brains, beef heart, peptone, sodium chloride, D-glucose, disodium hydrogen phosphate) at 37°C. After the initial purity check, one colony was taken and dissolved in 25 mL of liquid BHI. This liquid culture was incubated overnight until the stationary phase. This culture was diluted 20x in fresh BHI, and 7.5 mL of this inoculum was transferred to 20 mL glass ampoules. Two ampoules were added with 150 μL of bacteriophages from the beginning using a pipette. Two others were added with 150 μL of bacteriophages using the TAM admix ampoule injection system at different time points (between 1 and 3 h, corresponding to the early exponential phase). Finally, two ampoules without the addition of bacteriophages served as growth controls. After preparation, the samples were sealed and introduced into a TAM Air calorimeter (Waters/TA Instruments) that had been previously equilibrated at a temperature of 37°C ± 0.01°C. This device has eight measuring channels and eight slots for inert thermal references of the same heat capacity and conductivity as the samples, which were prepared with equal amounts of sterile PBS. Negative controls were prepared using two ampoules filled with uninoculated media. All measurements were performed in duplicates, and experiments were repeated twice.

### Calorimetry of thin biofilm

Eight plastic inserts were prepared with 250 μL of solution prepared with 100x diluted overnight cultures, as described above. The inserts were then placed in titanium calorimetry vials and sealed. Following closure, they were placed in the Calscreener calorimeter (Symcel Sverige AB), according to the manual 3-step equilibration procedure. The Calscreener was previously equilibrated at 37°C for at least 2 days. After 1 day of incubation and once heat production returned to baseline, the planktonic cells were removed by pipetting. The non-adherent cells were removed by washing with BHI media, and finally, the insert containing the biofilm was refilled with the same amount of medium with or without bacteriophages. The phage treatment medium contained 10% phage product diluted in BHI. The growth controls were made with BHI only. Sterility controls were made with uninoculated BHI. All measurements were performed in four replicates.

### Calorimetry of thick biofilm on nylon membrane

Previously sterilized nylon filters (1 cm × 3 cm–0.2 μm, Millipore, Burlington, MA, USA) were placed on BHI agar. A stationary-phase overnight culture of Staphylococci strains was spread on those filters using a 10 μL sterile inoculating loop. The plates with the biofilm were incubated at 37°C overnight. After biofilm formation under these conditions, the filters were transferred to fresh BHI agar until a mature biofilm state was achieved. Biofilms were considered mature when no visible changes could be seen in their size (surface and thickness on the filter) or appearance. This ensured that any changes detected in metabolic heat production were due to changes in metabolic activity induced by the antimicrobial treatment and not to further growth or extension of the biofilm on the membrane. All media and materials (including the cut nylon filters) were sterilized by autoclaving for 20 min at 121°C.

To detect biofilm heat production with IMC, mature biofilms grown on nylon filters were placed in 20 mL calorimetry glass vials (Waters/TA Instruments) containing slanted BHI agar. After sealing the ampoule, the sample was placed in the microcalorimeter (TAM3 - Waters/TA Instruments), and metabolic heat production was recorded in real-time. When metabolic heat production returned close to baseline, the vials were recovered from the calorimeter, and the same mature biofilms were exposed to bacteriophages. For this, 250 μL of phage product was applied to the biofilm until the solution was fully absorbed (within 5–10 min). After bacteriophage exposure, the nylon membranes bearing biofilms were transferred to fresh BHI agar vials to measure the heat flow again, following the same procedure. At the end of the experiment, the biofilms were heat-killed, and metabolic heat production was collected for the last time to establish a baseline signal. All measurements were performed in triplicate.

### Data analysis

Each experiment was performed in at least duplicate. The data analysis was performed using the statistical program R (R Core Team, 2019) and the grofit package (Kahm et al., 2010). Heat flow curves were integrated to obtain the heat over time curves. The Gompertz growth model was used to fit the heat curves and further calculate the maximal growth rate (*μ*), the lag phase (*λ*), and the maximum heat (Q max). When several overlapping peaks were observed, those peaks were convoluted with Fityk (Wojdyr et al., 2004) using the Pearson VII model. Then, each individual peak of the data was analyzed as described above.

## Results

Clear differences in phage product sensitivity were observed between planktonic cell cultures, thin biofilms, and thick biofilms (Table 1). Planktonic cells were the most affected, and a clear decrease of growth indicators *μ* and Q was observed in conjunction with an extension of the lag phase (*λ*). This effect on the lag phase was no longer visible for biofilm, as it had previously grown and already had a large active population of bacterial cells. With the increase in biofilm thickness, all effects were lost. All the details are provided in the specific sections.

**Table 1:** Growth parameter [growth rate (*μ*), lag phase duration (*λ*), total heat produced (Q)] calculated from the calorimetric data.

ConditionMicroorganism	Liquid culture*			Thin biofilm			Thick biofilm		
	% μ	Δ λ	% Q	% μ	Δ λ	% Q	% μ	delta λ	% Q
SE 49461	36.9 ± 9.9	12.2 ± 15.1	91.9 ± 4.0	49.2 ± 3.1	0.0 ± 0.4	110.3 ± 2.5	100.9 ± 22.7	1.7 ± 1.5	111.0 ± 32.4
SA 43300	30.2 ± 12.5	0.0 ± 0.5	24.2 ± 9.3	49.0 ± 7.0	0.0 ± 0.3	59.2 ± 7.8	123.1 ± 15.5	1.4 ± 0.2	108.7 ± 3.3
SA 25923	46.6 ± 27.8	13.5 ± 15.8	47.7 ± 13.5	70.7 ± 20.6	0.0 ± 1.5	78.0 ± 15.7	146.4 ± 14.2	0.0 ± 0.3	107.3 ± 3.9
SA 29213	97.0 ± 1.1	0.0 ± 0.2	101.9 ± 1.7	80.1 ± 4.0	0.4 ± 0.4	125.1 ± 9.9	ND	ND	ND
SAg 6,784	98.7 ± 2.9	0.1 ± 0.1	96.7 ± 1.2	ND	ND	ND	ND	ND	ND

SE, *Staphylococcus epidermidis*; SA, *Staphylococcus aureus*; Sag, *Streptococcus agalactiae*; numbers refer to the ATCC or DSM culture collection number. The growth rate (μ) and the total heat (Q) are expressed as a percentage of the growth control. The lag phase duration (λ) is expressed in additional lag hours. *Only data for phage addition at time = 0 are shown.

### Effect of bacteriophages on planktonic cells in liquid culture

**Figure 1:**
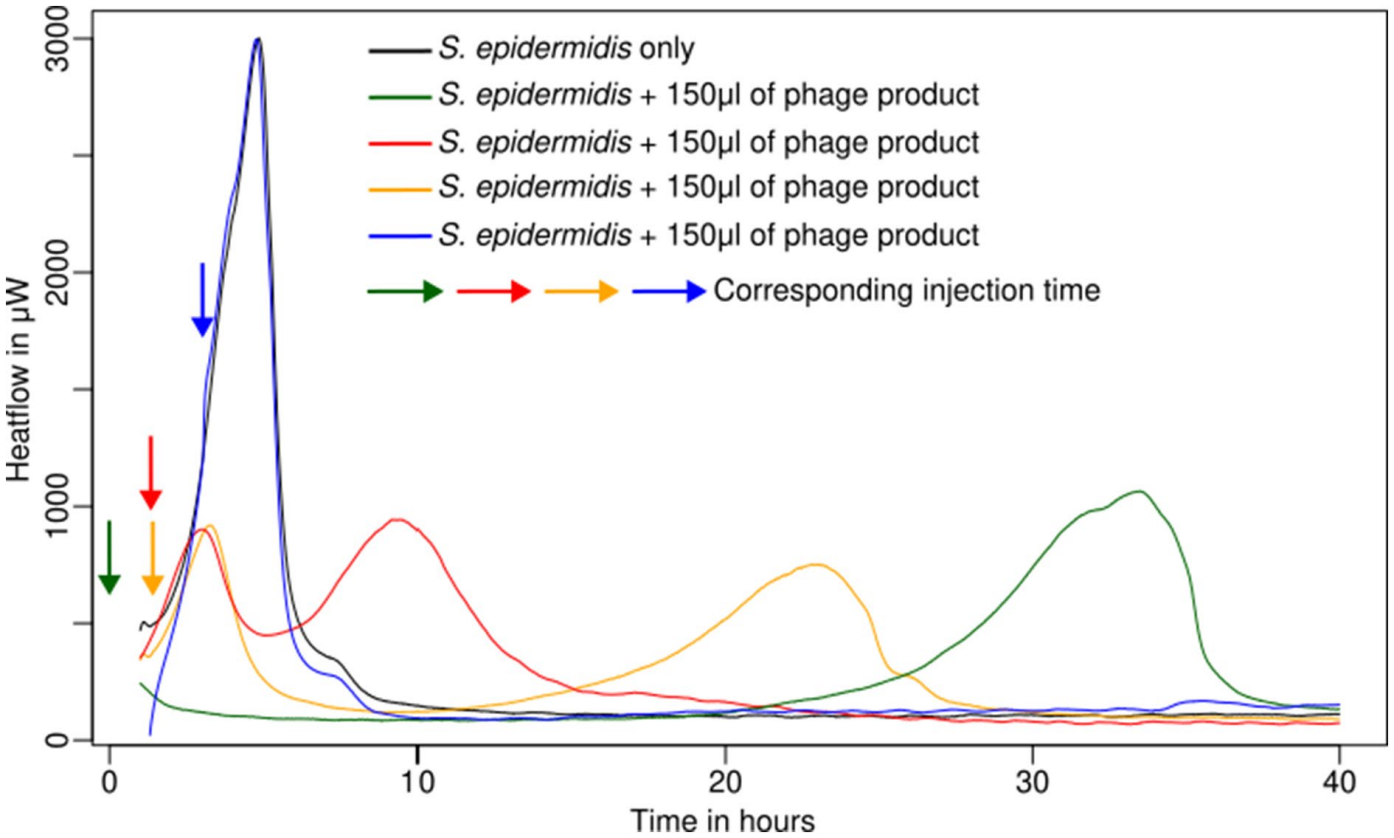
Metabolic activity of planktonic cells of *S. epidermidis* ATCC 49461 in liquid culture monitored using IMC.

The effect of the bacteriophages was tested for bacteria in planktonic form by adding the phage product from the beginning or later in the early exponential phase (between 1 and 5 h; corresponding to 300 and 800 μW), with an injection system, in comparison with growth without inhibition (see Table 1 and Figure 1).

*Staphylococcus epidermidis* (ATCC 49461) and *S. aureus* (ATCC 43300, ATCC 25923) appeared sensitive to the phage product showing a rapid decline in metabolic heat production when the phage was added at the beginning of the experiment or early enough (up to 2 h after measurement started) (Table 1; see example in Figure 1). On the other hand, *S. aureus* ATCC 29213 and *Streptococcus agalactiae* (*S. agalactiae*) DSM 6784 displayed complete resistance (Table 1) irrespective of the phage addition time. Looking in more detail, delaying phage addition significantly reduced their inhibitory effect (see example in Figure 1). This was quite clear for all sensitive Staphylococci. In addition, once the bacterial load was too high (and consequently multiplicity of infection (MOI) too low), the addition of phages did not show any effect on the metabolic heat. Overall, in our conditions, injection after 2 h still produced a weak inhibition; however, after 3 h, no effect was visible for any of the tested strains.

In two cases (*S. epidermidis* ATCC 49461 and *S. aureus* ATCC 25923), regrowth could be observed after 15–30 h following initial phage-induced suppression, which occurred immediately after phage injection (see example in Figure 1). We assumed that phage-resistant Staphylococci have emerged or that the bacteriophages have been inactivated, allowing bacterial growth to resume using the remaining nutrients.

### Effect of bacteriophages on thin biofilms

**Figure 2:**
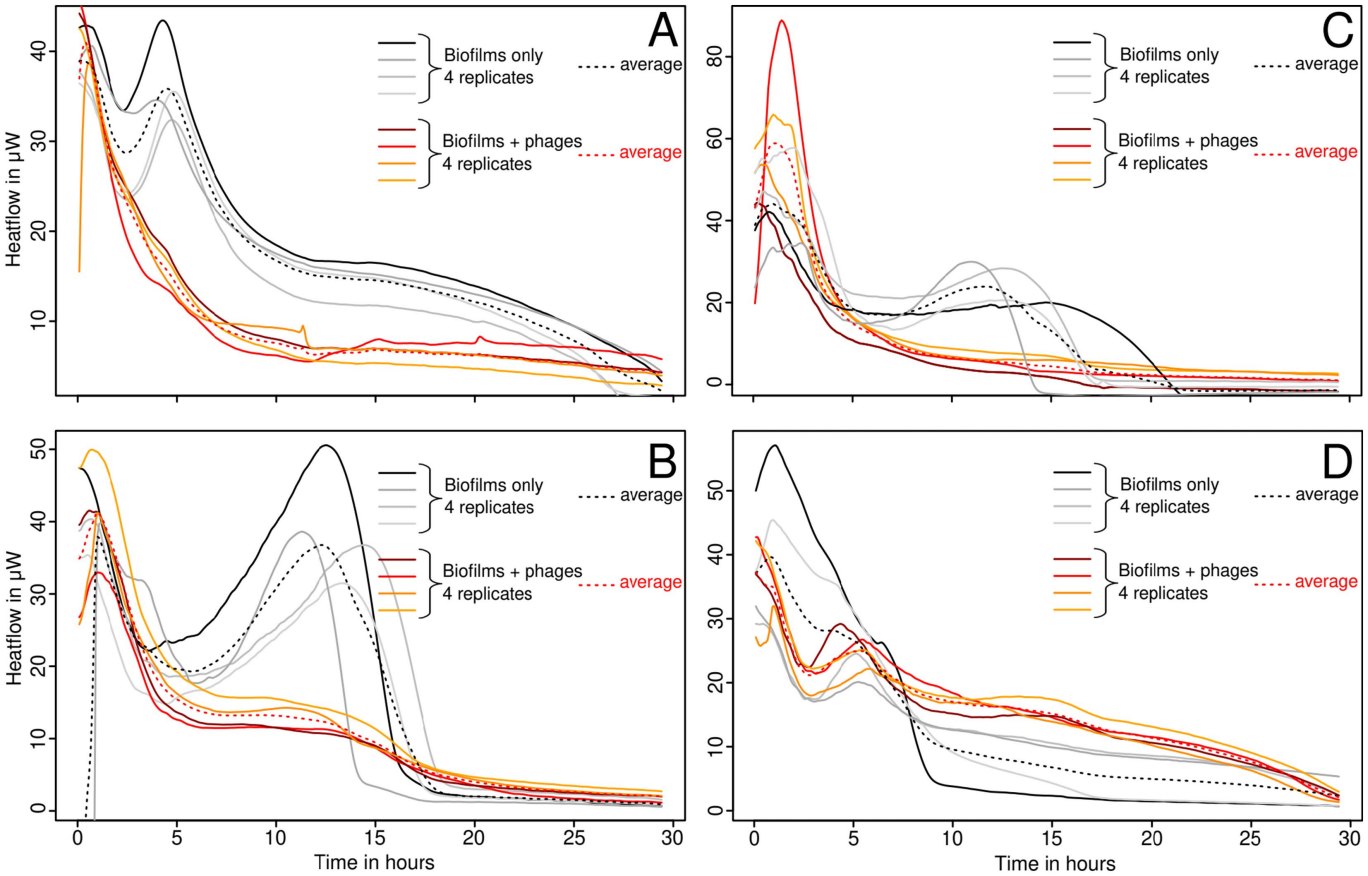
Growth of thin biofilms of *S. epidermidis* ATCC 49461 **(A)**, *S. aureus* ATCC 43300 **(B)**, ATCC 25923 **(C)**, and ATCC 29213 **(D)**, monitored using IMC with and without bacteriophages.

Thin biofilms grown in calorimetry inserts were analyzed after removal of the planktonic cells and non-adherent cells. For this part, we focused on the strains that proved to be sensitive in the first part of the experiment (*S. epidermidis* ATCC 49461, *S. aureus* ATCC 43300 and 25,923) and the resistant one (ATCC 29213) as a control (Figure 2).

For *S. epidermidis*, a lower maximum growth rate (*μ*) was observed with phages, although the peak heat production (Q max) was higher (Figure 2A). This suggests that a high metabolic activity was sustained for a longer period, likely due to slower nutrient depletion in the medium. *S. aureus* ATCC 43300 (Figure 2B) and *S. aureus* ATCC 25923 (Figure 2C) exhibited significant inhibition of both growth and heat production, indicating effective phage activity. Meanwhile, *S. aureus* ATCC 29213 (Figure 2D) showed no significant change, consistent with its previously observed resistance to phages in liquid cultures (Figure 1).

### Effect of bacteriophages on thick biofilms

**Figure 3:**
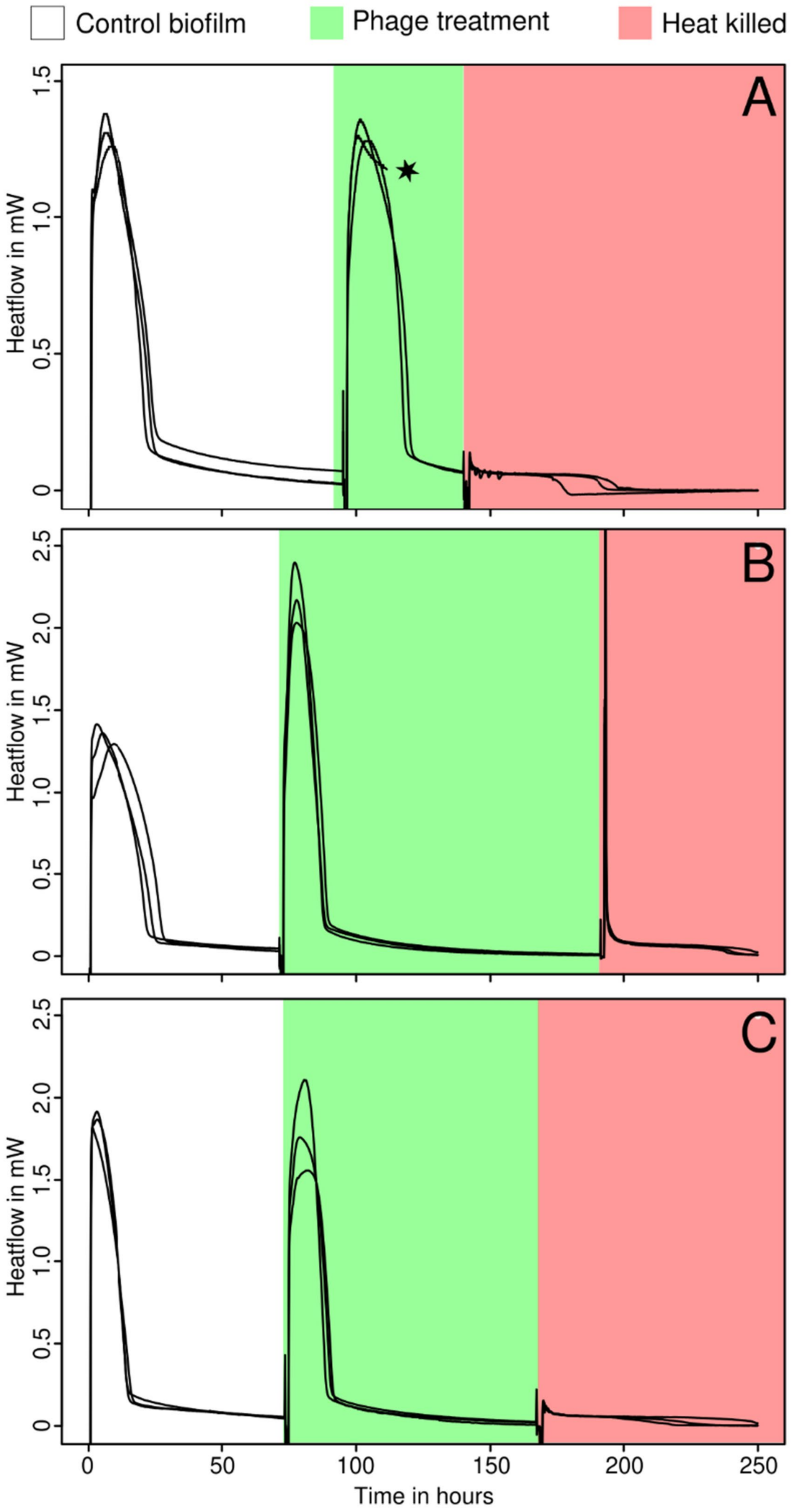
Growth of thick biofilms of *S. epidermidis* ATCC 49461 **(A)**, *S. aureus* ATCC 25923 **(B)**, and ATCC 43300 **(C)** monitored using IMC before (white) and during the addition of bacteriophages (green) and after heat killing (red). The star indicates that some data were omitted due to technical difficulties or methodological issues.

For this part of the experiment, we only analyzed the three sensitive *Staphylococcus* strains. In this part of the experiment, the same biofilm was measured both with and without phage insertion and in new BHI medium. No significant effect from bacteriophages was detected on any of the strains tested. The biofilm was most likely too thick and therefore resistant. No heat production was observed after heat killing (Figure 3). It must be noted that the growth indicator (*μ*, *λ*, and Q) used here reflects more the medium consumption rather than the net growth of the biofilm. Still, for ease of comparison, these indicators were calculated in the same manner.

## Discussion

Observation of the heat flow curves reveals interesting phenomena such as the appearance of secondary peaks after returning to baseline for the planktonic cells in liquid medium, the prolonged maintenance of stable metabolic levels in thin biofilms, and finally, the complete resistance of thick and mature biofilms to isothermal microcalorimetry. Indeed, in planktonic cultures, resistant bacterial mutants might be selected, enabling regrowth as long as the nutrients are not depleted. Furthermore, recent studies outlined the fact that phages can lead to the production of persisters host cells (Fernández-García et al., 2024). This certainly deserves more attention in future studies. For thin biofilms, with thicknesses usually ranging between 9 and 40 μm, according to the literature, which uses closely similar preparations (Shi et al., 2016; Di Stefano et al., 2009; Shukla and Rao, 2013; Liu et al., 2015), it appears that part of the biofilm remains active. Still, thin biofilms are unable to regrow and disperse in the liquid phase. Most likely, the phage is preventing microbes from dispersing in the liquid medium, but cannot penetrate the entire biofilm and thus eliminate all microbes. Staphylococci protected in a biofilm can then survive using the remaining nutrients from the medium. In our study, mature biofilms appeared particularly resistant. When prepared on membranes, Staphylococci biofilms have a thickness varying between 270 and 311 μm; however, some studies have reported thicknesses up to 700 μm (Agostinho et al., 2011; Singh et al., 2010; Chatterjee et al., 2014). Therefore, it is not surprising that several studies have already shown that mature biofilms are orders of magnitude more resistant to several antibiotics (Nickel et al., 1985; Mah and O’Toole, 2001). Similarly to previously published studies and reviews, we can safely assume that the same principle also applies to the efficacy of bacteriophages against biofilms (Abedon, 2023). This may be due to several factors such as the barrier against chemical and physical substances provided by to the exopolysaccharide matrix of the biofilm and thus the inability of the phages to penetrate, the binding and trapping of phage on the EPS matrix, the acquired resistance mechanisms expressed only in biofilms or a metabolism that is too slow to allow the phages to reproduce sufficiently (Abedon, 2023). From a more clinical perspective, thick biofilms are a significant factor in inhibiting the healing of chronic wounds, and optimal management to remove such biofilms requires surgical debridement as an additional step (Scalise et al., 2015). While debridement remains the most cost-effective strategy for reducing biofilm, it cannot completely eradicate it. Continued debridement is essential to maintain the biofilm in a weakened state, which allows adjunctive treatments such as phages or antibiotics to play a critical role in the healing of chronic wounds (Wolcott et al., 2009; Anghel et al., 2016). These adjunctive therapies help disrupt biofilm formation and improve the overall effectiveness of wound care (Chan and Abedon, 2012). Although more data are required to support this statement, it appears that the combination of debridement and phages should be further investigated as a therapeutic option (Chan and Abedon, 2012; Parracho et al., 2012).

In the context of phage therapy, the present data and previous studies show that the use of IMC has proven to be an effective method for analyzing bacteriophage-bacteria interactions and their kinetics, as it proved to be faster method than traditional cultures (i.e., agar overlay requiring up to 24 h) without requiring additional work. Compared to optical density-based methods, IMC is also suitable for analyzing opaque liquid and solid media, which has proven effective for testing thin and thick biofilms without the need for destructive methods (Stewart and Franklin, 2008). In addition, the growth and inhibition kinetics can be studied using heat curve analysis as described before (Braissant et al., 2013). Still, a thorough comparison of all current methods to assess phages with IMC should be conducted in order to assess the potential of this technique.

It must also be noted that IMC has some limitations. In particular, it uses sealed airtight vials that limit the amount of oxygen in the system. In addition, as oxygen diffuses poorly in aqueous solution, this may lead to severe limitations when using strictly aerobic microbes (Maskow et al., 2014). This was not the case for the Staphylococci and Streptococci used here, which are capable of fermenting a wide range of substrates present in the medium used. Another important limitation of isothermal microcalorimetry is that most of the heat generated during growth comes from catabolic reactions. The synthesis of phages, like other anabolic processes, does not release heat (or a negligible amount – see the study by Battley, 1998; Battley, 1992; Kell and Battley, 1987) and remains invisible despite its cost to the cell. Therefore, combining IMC with ATP measurements, flow cytometry, protein assays, or PFU count might be valuable (Braissant et al., 2020; Braissant et al., 2015; Morais et al., 2014). Similarly, the calorespirometric approach may also be of interest, as well as the heat per O_2_ and the heat per CO_2_, which may provide additional information. To the best of our knowledge, calorespirometry has not been investigated with bacteriophages yet. In addition to the limitations imposed by IMC itself, the following limitations of the study should also be taken into account. Firstly, only one strain of *Streptococcus* was investigated in detail and found to be resistant (preliminary studies have also shown that other dental strains were not sensitive to phage cocktails – data not shown). However, the limited number of strains tested does not allow for any conclusions on the efficacy of Streptococci. Similarly, with respect to chronic wounds, Gram-negative pathogens such as *Pseudomonas aeruginosa* should also be included in future studies as they can represent an important proportion of causative pathogens in diabetic foot infections and various ulcer-related infections (Rahim et al., 2017; Ramakant et al., 2011; Kirketerp-Møller et al., 2008). This is certainly a limitation of the current study, especially as phages against *Pseudomonas aeruginosa* are indeed available in online pharmacies. Finally, it should be noted that infection by both pathogens is also common, emphasizing the need to test several phage products simultaneously (Ibberson et al., 2017; Lichtenberg et al., 2023). From a more technical standpoint, during experiments with planktonic cells, the injection system may have altered the metabolism by mixing the medium and resuspending cells that otherwise would have sedimented; thus, creating a potentially more favorable environment for bacterial growth, we believe that the effect is extremely limited and did not influence the results. Similarly, for a thick biofilm, the handling with sterile tweezers might have altered the surface of the biofilm. We estimate that <1% of the surface of the biofilm might have been altered during the transfer of the filters to fresh medium. This may have been due to the presence of an entry window for phages in the Fersici cocktail. Still, all biofilms proved to be resistant, which supports that the idea that handling did not affect the results.

## Conclusion

Bacteriophages appear to be a valid solution to the growing resistance to antibiotics as they are an effective agent for killing bacteria in planktonic cells and thin biofilms, such as those found in many skin infections. With increasing biofilm thickness and maturation, it is likely that more aggressive measures (cleaning and debridement) need to be taken prior to the application of phages, as our study demonstrated that thick biofilms of sensitive microbes are not affected by phages. However, it should be noted that antibiotics face similar, if not greater, challenges in treating thick biofilms. Thus, emphasizing the need for further studies, especially *in vivo* studies combining debridement and phage therapy. In addition, a better understanding of bacterial metabolism when infected with phages would be desirable, as IMC is rather insensitive to phage production. The use of incorporation assays, such as stable isotope labeling or substrate analogue labeling, may provide some significant insights into phage production and energetic costs (Braissant et al., 2020; Hatzenpichler et al., 2014). In addition, the use of substrate analogues would allow tracking the phages using fluorescent markers through click chemistry.

## Data Availability

The raw data supporting the conclusions of this article will be made available by the authors, without undue reservation.

## Footnotes

^1^ https://mybacteriophage.net/products/fersisi-bakteriophagen-1-box-5-ampullen-x-10ml
